# Mitofusin 2, a key coordinator between mitochondrial dynamics and innate immunity

**DOI:** 10.1080/21505594.2021.1965829

**Published:** 2021-09-04

**Authors:** In Soo Kim, Prashanta Silwal, Eun-Kyeong Jo

**Affiliations:** a Department of Microbiology, Chungnam National University College of Medicine, Daejeon, Korea; b Infection Control Convergence Research Center, Chungnam National University College of Medicine, Daejeon, Korea

**Keywords:** Mitofusin-2, mitochondrial dynamics, innate immunity, infections

## Abstract

Remodeling of mitochondrial dynamics and mitochondrial morphology plays a pivotal role in the maintenance of mitochondrial homeostasis in response to pathogenic attacks or stress stimuli. In addition to their role in metabolism and energy production, mitochondria participate in diverse biological functions, including innate immune responses driven by macrophages in response to infections or inflammatory stimuli. Mitofusin-2 (MFN2), a mitochondria-shaping protein regulating mitochondrial fusion and fission, plays a crucial role in linking mitochondrial function and innate immune responses. In this article, we review the role of MFN2 in the regulation of innate immune responses during viral and bacterial infections. We also summarize the current knowledge on the role of MFN2 in coordinating inflammatory, atherogenic, and fibrotic responses. MFN2-mediated crosstalk between mitochondrial dynamics and innate immune responses may determine the outcomes of pathogenic infections.

## Introduction

Mitochondrial dynamics are regulated by mitochondrial fusion and fission and the formation of mitochondrial networks. In turn, these processes modulate various biological functions, including mitochondrial metabolism, redox balance, and cell death [1]. Mitochondrial fusion and fission are regulated by a group of guanosine triphosphatases (GTPases) [2,3]. Specifically, mitochondrial fusion is controlled by the GTPases mitofusin-1 (MFN1) and mitofusin-2 (MFN2), which are located in the outer mitochondrial membrane (OMM), and by the GTPase optic atrophy 1 in the inner mitochondrial membrane. Mitochondrial fission is regulated by the dynamin-related protein 1 (Drp1) through interaction with other OMM proteins, such as MiD51 and MiD49 [4,5]. These proteins are essential for mitochondrial quality control and homeostasis, as well as the regulation of numerous biological processes, including immune responses [6,7].

Innate immune responses involving sensing of microbes and generation of antimicrobial effectors are the primary defense in response to pathogenic attacks and damage signals. Accumulating evidence suggests that multilayered molecular crosstalk between innate immune signaling and mitochondrial dynamics regulates the outcomes of pathogenic infections. For example, innate immune signaling triggered by retinoic acid-inducible gene I-like receptors (RLRs) induces remodeling of the mitochondrial network [8]. RLR activation by viral RNA promotes mitochondrial elongation to enhance the interaction between mitochondrial antiviral signaling protein (MAVS; also known as IPS-1, VISA, or CARDIF) and other signaling proteins of the endoplasmic reticulum (ER) [8]. Toll-like receptor (TLR) 4 signaling has been linked to Drp1-mediated mitochondrial fission and macrophage-mediated inflammatory responses [9], which orchestrate the primary immune responses during infection [10]. In particular, MFN2 seems to be a critical player in linking mitochondrial dynamics, autophagy (xenophagy and mitophagy), and innate immune responses against viruses, bacteria, and parasites [11–14]. Recent findings also suggest that MFN2 regulates inflammatory, atherogenic, and fibrotic responses in a context-dependent manner [15–17]. In this article, we summarize the current knowledge on the role of MFN2 in the crosstalk between mitochondrial dynamics, innate immunity, and immunometabolism in macrophages during infection.

## Innate immunity: RLR signaling, inflammasome, and immunometabolism

Innate immune responses are the primary immune defense in response to infectious agents. Innate immune cells, including macrophages and dendritic cells, express various pattern-recognition receptors (PRRs), which recognize pathogen-associated molecular patterns and damage-associated molecular patterns derived from microbial cells and damaged cellular components. Activation of TLRs, nucleotide-binding oligomerization domain (NOD)-like receptors (NLRs), and other PRRs can trigger complex intracellular signaling pathways. These innate immune pathways are regulated by various kinases, phosphatases, signaling enzymes, adaptors, and protein scaffolds [18–20]. In response to these pathways, antimicrobial peptides and inflammatory cytokines are released to counterattack the invading pathogens. Innate immune responses are tightly regulated to prevent tissue damage due to the overactivation of inflammatory responses [18,19]. In addition to serving as the primary defense, innate immunity is also crucial for the initiation of adaptive immune responses, which protect from subsequent infections by the same pathogen [18,19,21,22]. Moreover, well-characterized immune functions are interconnected to metabolic remodeling in the innate immune cells to shape effector functions and inflammatory responses upon the challenge of diverse pathogens [23–25]. Recent studies have highlighted the involvement of mitochondrial dynamics in the regulation of immunometabolism to influence innate immune functions, as we discuss in the latter part of this review. In this section, we have divided our discussions of RLR signaling, inflammasomes, and immunometabolism, to better explore the inter-related function of MFN2 in the context of these immune response paradigms.

### RLR signaling

Cytoplasmic RNA from RNA viruses can activate intracellular RNA-specific PRRs, such as retinoic acid-inducible gene 1 (RIG-I) and melanoma differentiation-associated gene 5 (MDA5). Recognition of viral RNA fragments by RIG-I and MDA5 initiates signal transduction through MAVS on the surface of mitochondria. MAVS, a key adapter protein of RLR signaling, contains three domains; amino-terminal caspase activation and recruitment domain (CARD), a proline-rich central domain, and a carboxy-terminal transmembrane domain to anchor at the OMM. Upon RIG-I or MDA5 activation, either PRR can bind MAVS through CARD-CARD interactions and induces the formation of large MAVS aggregates, thereby propagating downstream signaling cascade that activates the cytoplasmic kinases, such as tumor necrosis factor receptor-associated factor family member-associated nuclear factor κ-light-chain-enhancer of activated B cells binding kinase 1 (TBK1) and IKB kinase-ε [26–28]. The activation of protein kinases, in turn, phosphorylate the transcription factors interferon regulatory factor (IRF) 3 and nuclear factor κ-light-chain-enhancer of activated B cells (NF-κB), both of which are crucial transcriptional factors to activate a variety of innate immune-related genes, including type I interferons (IFNs) and pro-inflammatory cytokine genes to regulate antiviral and inflammatory responses during viral infection [26,27]. How an MFN2-MAVS interaction at OMM influences mitochondrial inner compartment is less well understood due to a topologic gap between the OMM and inner mitochondrial membrane (IMM). The IMM protein prohibitins are mitochondrial scaffold proteins involved in mitochondrial signal transduction and homeostasis [29]. Future work is required to understand whether prohibitins may bridge MFN2/MAVS with IMM as a part of prohibitin interactome assembly that is crucial for mitochondrial functions [29].

In addition, RLR-induced antiviral signaling is regulated through extensive post-translational modifications at each step via multiple ubiquitin E3 ligases, deubiquitinases, protein kinases, and phosphatases [30–35]. The detailed mechanisms underlying post-translational modification of RLR-triggered signaling are beyond the scope of this review. The antiviral immune responses are tightly regulated to prevent the uncontrolled secretion of IFNs [36,37]. Emerging evidence suggests that mitochondria are essential for the activation of RLR signaling in response to viral infections. Through complex host-viral interactions, viruses can manipulate the mitochondrial machinery of the host to suppress innate immune activation [38–40].

### Inflammasome complex

The inflammasome complex is a large intracellular multiprotein platform that activates caspase-1. In turn, caspase-1 proteolytically cleaves cytosolic pro-interleukin (IL)-1β and pro-IL-18, and the subsequent release of pro-inflammatory cytokines results in pyroptotic cell death. NLRP3 (NOD-like receptor protein 3) is the most extensively studied inflammasome. Recent evidence suggests that post-translational modifications, protein-protein interactions, and spatiotemporal regulatory mechanisms regulate activation of the NLRP3 inflammasome [41,42]. Furthermore, crosstalk between the ER and mitochondria in mitochondria-associated membranes (MAMs) is essential for inflammasome activation [43]. MFN2-induced tethering between the ER and mitochondria is critical for the formation of MAMs to promote inflammasome-mediated cellular defense [43–45]. Aberrations in MAMs have been implicated in various pathological processes. In particular, alterations in MAMs increase the levels of mitochondrial reactive oxygen species (ROS) and promote intracellular translocation of mitochondrial components, thereby promoting chronic inflammation and metabolic rewiring [43,44].

### Immunometabolism in innate immune cells

Innate immune signals, such as TLR ligands, trigger the differentiation of macrophages into pro-inflammatory M1 macrophages by enhancing aerobic glycolysis and suppressing the tricarboxylic acid cycle. In M1 macrophages, succinate enhances ROS generation, thereby stabilizing hypoxia-inducible factor 1α (HIF-1α) and promoting aerobic glycolysis, IL-1β secretion, and M1 polarization [46–49]. In the early phases of inflammation, an immunometabolic shift to aerobic glycolysis, known as the Warburg effect, is required for cells to fulfill their high energy demands [46,50]. At a later stage of inflammation, metabolic reprogramming in immune cells increases mitochondrial oxidative phosphorylation and fatty acid oxidation (FAO), both of which are required for inflammation resolution and tissue repair [51]. However, recent findings suggest that FAO promotes inflammasome activation [24,52]. Moreover, increased glucose utilization via aerobic glycolysis and the Akt-mammalian/mechanistic target of rapamycin complex 1 pathway is required for lipid metabolism and FAO in activated M2 macrophages and type 2 T helper cells [52–54]. Accumulating evidence suggests that the mitochondrial dynamics shaped by fusion and fission events are intimately linked to the regulation of immunometabolism, thereby leading to diverse outcomes in various immune cell functions [55–58]. Future studies are needed to dissect the immunometabolic profiles in each type of immune cell at different stages of infection and inflammation and elucidate how mitochondrial dynamics interconnect with the molecular networks of immunometabolism to determine infection outcomes.

## Components regulating mitochondrial dynamics

Mitochondrial fusion and fission are critical regulators of mitochondrial morphology and function [59]. Mitochondrial fusion is essential for the exchange of metabolites and DNA between neighboring mitochondria, the delivery of metabolites to the cell core from the periphery, and the maintenance of the protein stoichiometry of the mitochondrial DNA replisome [7,60–62]. The GTPase activity of MFN1 and MFN2 is responsible for membrane tethering, fusion, and mitochondrial calcium influx [61,63]. Mitochondrial fission often results in uneven daughter mitochondria; one daughter unit is eliminated by mitophagy [64]. Fission is essential for the segregation of damaged mitochondria and the maintenance of energy demands in different subcellular regions [64,65]. In addition, fission facilitates apoptosis during extreme levels of cellular stresses through the segregation of damaged mitochondria [65]. Upon selective stresses, including UV irradiation or actinomycin D treatment, mitochondria form a highly interconnected network depending on the mitochondrial inner membrane protein stomatin-like protein 2, but not MFN2 [66]. This response may contribute to an adaptive pro-survival mechanism against selective stresses in the cells [66]. In response to a variety of infectious stresses, mitochondrial dynamics may control the immune activation through multiple mechanisms, which are discussed in the following sections.

The coordination between fusion and fission is a critical regulator of mitophagy and apoptosis, thereby controlling the balance between cell survival and cell death [67]. Despite this, it remains to be determined which signaling pathways play a specific role in the crosstalks between mitochondrial dynamics and other mitochondrial functions. Recent studies showed that the cytoplasmic DNA sensor cyclic guanosine monophosphate -adenosine monophosphate (AMP) synthase (cGAS)/protein stimulator of interferon genes (STING) signaling is involved in a variety of roles in mitochondria, i.e., apoptosis, autophagy, sensing of mitochondrial DNA, and inflammatory signaling in different settings of physiological and pathological conditions [68–71]. Previously, MFN1 deficiency suppresses STING pathway activation to decrease IFN-β induction as well as diminished signaling activation of TBK1 and IRF3 [72]. Given the central role of cGAS/STING signaling in innate and mitochondrial functions, further studies are needed to clarify the molecular links between cGAS/STING signaling and mitochondrial dynamics.

## The role of MFN2 in innate immune responses during infection

### Overview of MFN2 function

MFN2 participates in the initial step of mitochondrial fusion [73,74]. In humans, MFN2 contains 757 amino acid residues. It is a nuclear-encoded, dynamin-like GTPase harboring an N-terminal GTPase domain and a C-terminus exposed to the cytosol. The GTPase domain of MFN2 hydrolyzes GTP, promoting MFN oligomerization and OMM fusion between adjacent mitochondria [75,76]. Mammalian MFN2 shares ~ 80% sequence similarity with MFN1 and is essential for OMM fusion. Although the GTPase activity of MFN2 is lower than that of MFN1, MFN2 has a higher GTP affinity [63]. MFN2 and MFN1 contribute to tethering between the ER and mitochondria [63]. MFN2 also regulates PTEN-induced kinase 1 (PINK1)/Parkin-mediated mitophagy [77]. Upon mitochondrial membrane depolarization, MFN2 interacts with Parkin in a PINK1-dependent manner. Upon phosphorylation by PINK1 [77], MFN2 is ubiquitinated by Parkin; subsequently, MFN2 is degraded through the action of p97, a member of adenylpyrophosphatase associated with diverse cellular activities, that accumulates in mitochondria and is required for Parkin-induced mitophagy [78]. Through this process, MFN2 promotes the removal of damaged or senescent mitochondria [62].

Besides its fundamental role in mitochondrial fusion, MFN2 also regulates various other biological processes, such as cell proliferation and cell death [62,79,80]. Because MFN2 is required for axonal mitochondrial transport in neurons, dysfunction of MFN2 has been associated with several neurodegenerative diseases, such as Charcot-Marie-Tooth disease and Alzheimer’s disease [81–83]. Moreover, dysregulated MFN2 expression and function have been implicated in type 2 diabetes mellitus [84] and atherosclerosis [85,86]. In gastric cancer, MFN2 has been shown to exert antitumor effects [80]. By contrast, patients with breast cancer expressing low MFN2 levels show poor prognosis, at least partly due to the ability of MFN2 to inhibit mTORC2 [87]. Future studies are required to uncover the role of MFN2 in different disease conditions, including cancers.

### MFN2 and innate immune responses during viral infections

Numerous recent studies have shown that MFN2 is a key regulator of innate immune responses during viral infections. Earlier studies reported that MFN2 inhibited antiviral immune responses by interacting with MAVS [11]. Additionally, MFN2 overexpression inhibited RLR signaling and IRF3 expression; by contrast, MFN2 deficiency enhanced IFN-β secretion and suppressed viral replication [11]. Structural evidence suggests that MFN2 interferes with MAVS homotypic oligomerization to alter the active conformation of MAVS [88]. More recently, MFN2 was found to regulate corticosterone-mediated boosting of influenza virus (H1N1) replication by interacting with MAVS. MFN2 mediates the recruitment of E3 ligase synoviolin 1, thereby promoting MAVS polyubiquitination [89]. Of note, MFN1 may play an opposite role by interacting with MAVS during viral infection. MFN1 binds MAVS and promotes the production of antiviral IFNs [90]. Further evidence suggests that MAVS interacts with MFN1 to facilitate ER-mitochondrial association, thereby enhancing the downstream signaling of MAVS [8]. Cytomegalovirus-induced mitochondrial fragmentation is one of the immune-escaping strategies viruses employ to inhibit the binding of MAVS to STING1 and suppress antiviral immune responses [8]. Together, these data suggest that MFN1 and MFN2 interact with MAVS to fine-tune innate immune responses during viral infection.

However, it remains unclear how MFN levels are regulated during viral infection. Triggering receptor expressed on myeloid cells 1 (TREM1) has been identified as one of the upstream signaling molecules regulating MFN2 expression. During infection with human immunodeficiency type 1 (HIV-1), MFN1 and MFN2 are upregulated in macrophages in a TREM1-dependent manner. The decreased expression of anti-apoptotic proteins and MFNs is associated with increased levels of apoptosis in macrophages due to alterations in mitochondrial membrane potential (MMP; ΔΨ(m)) [91]. Thus, TREM1-dependent MFN2 upregulation and inhibition of apoptosis may contribute to the ability of HIV-1 to survive within host cells [91].

Interestingly, MFN1 and MFN2 regulate host defenses against the dengue virus and are cleaved by dengue virus protease NS2B3. During dengue virus infection, MFN1 is essential for efficient antiviral signaling, whereas MFN2 maintains MMP to inhibit cell death [92]. Thus, dengue virus-mediated cleavage of MFNs suppresses mitochondrial fusion and virus-induced cytopathy [92]. Future studies are warranted to reveal the molecular mechanisms by which MFNs regulate antiviral responses and host cell survival in response to different viruses. The intracellular signaling events regulating the expression of MFNs during infection also merit further investigation.

MMP is essential for MAVS-induced antiviral responses [93]. MFN2 is required for MMP homeostasis and IL-1β secretion after infection with RNA viruses, including influenza, measles, or encephalomyocarditis virus (EMCV) [94]. Conversely, overexpression of the inner mitochondrial membrane protein uncoupling protein 2 alters the MMP and reduces IL-1β secretion during infection. Mechanistically, MFN2 binds NLRP3 to promote IL-1β secretion after infection with influenza virus and EMCV [94]. However, it is unclear whether MFN2 is involved in activating the conventional NLRP3 inflammasome complex; whether MFN2 levels affect host defense and pathogenesis during viral infection also remains unclear. Several roles of MFN2 in innate immune responses during viral infection are summarized in Table 1.

**Table 1.** The role of MFN2 in innate immune responses during infection

Pathogen	Model	Effect	Mechanism	Ref.
***Viral infection***				
EMCV, Measles, VSV	HEK 293T cells; MEFs	Inhibition of RLR-induced antiviral signaling	Interaction with carboxyl-terminal MAVS	[11]
Influenza virus A (H1N1)	A549 cells; Stress mice model	Inhibition of RLR-induced antiviral signaling	Interaction with and degradation of MAVS	[89]
EMCV, Influenza virus, Newcastle disease virus, SeV, Sindbis virus, VSV	HEK 293T cells; MEFs	No effects	ND for MFN2; MFN1 interacts with MAVS and activates type I IFN signaling	[90]
Cytomegalovirus	HeLa; HEK 293T cell	No effects	ND for MFN2; MFN1 interacts with MAVS and activates type I IFN signaling	[8]
Human immunodeficiency virus1	Human monocyte-derived macrophages	Increase in viral reservoir	Inhibition of BCL2L11-mediated MMP disruption by TREM1	[91]
Dengue virus	A549 cells	Attenuation of virus-induced cytopathic effects	Suppression of caspase 3 activation and MMP disruption	[92]
EMCV, SeV, VSV	HEK 293T cells; *Mfn1* and *Mfn2* double mutant MEFs	Induction of RLR-induced antiviral responses	Enhancement of adequate MMP and rearrangement of MAVS	[93]
EMCV, Influenza virus, Measles	BMDMs; HEK 293T cells; J774A.1 mouse macrophages	Activation of inflammasome	Interaction with NLRP3 and MAVS complex	[94]
***Bacterial infection***				
ESAT-6, Mtb lysate	PBMCs; THP-1 cells	Activation of inflammasome	Interaction with NLRP3	[95]
*Aeromonas hydrophila, Listeria monocytogenes*, LPS, Mtb	BMDMs; *Mfn2*-cKO mice; L-929 cells; PMs	Induction of antibacterial responses and antigen processing	Increase in mitochondrial ROS and activation of ERK1/2, p38, and NF-κB	[12]
BCG, *L. monocytogenes*, Mtb,*Mycobacteroides abscessus*	BMDMs; *Mfn2*-cKO mice; PMs; RAW264.7 cells	Enhancement of aerobic glycolysis and xenophagy	Induction of mitochondrial ROS and HIF-1α	[14]
Mtb	BMDMs; RAW264.7 cells	Increase in bacterial growth	Inhibition of MMP disruption and apoptosis	[101]
***Parasitic infection***				
*Leishmania donovani*	Golden hamsters; PMs from BALB/c mice; RAW264.7 cells	Enhancement of antiparasitic microRNAs	Destabilization and compartmentalization of miRNP by mitochondria-ER tethering	[13]

BCG, *Mycobacterium bovis* bacillus Calmette-Guérin; BCL2L11, B-cell lymphoma 2 like 11; BMDM, bone-marrow-derived macrophage; EMCV, encephalomyocarditis virus; ER, endoplasmic reticulum; ERK, extracellular signal-regulated protein kinase; ESAT-6, 6-kDa early secreted antigenic target; HIF-1α, hypoxia-inducible factor 1 alpha; IFN, interferon; LPS, lipopolysaccharide; MAVS, mitochondrial antiviral-signaling protein; MEF, mouse embryonic fibroblast; Mfn1, mitofusin 1; Mfn2, mitofusin2; *Mfn2*-cKO mice, *Mfn2^fl/fl^:LysM^c/c^* mice; miRNP, microribonucleoprotein; MMP, mitochondrial membrane potential; Mtb, *Mycobacterium tuberculosis*; ND, Not detected; NF-κB, nuclear factor kappa-light-chain-enhancer of activated B cells; NLRP3, nucleotide-binding and oligomerization domain-like receptor pyrin domain-containing protein 3; PBMC, peripheral blood mononuclear cell; PM, peritoneal macrophage; RLR, retinoic acid-inducible gene-I-like receptors; ROS, reactive oxygen species; SeV, Sendai virus; TREM1, triggering receptor expressed on myeloid cells 1; VSV, Vesicular stomatitis virus

### MFN2 and innate immune responses during bacterial infection

Although MFN2 signaling has been mostly studied in the context of viral infection, recent studies suggest a role for MFN2 in regulating the interactions between bacteria and host cells. MFN2 was found to be upregulated in peripheral blood mononuclear cells of patients with active tuberculosis [95]. In addition, mycobacterial antigen 6-kDa early secreted antigenic target and *Mycobacterium tuberculosis* lysate enhanced the interaction between MFN2 and NLRP3, promoting assembly of the inflammasome complex and the production of IL-1β [95]. MAMs formed through tethering between the ER and mitochondria act as a scaffold for NLRP3 inflammasome activation [43]. MFN2 mediates the formation of MAMs by promoting tethering between the ER and mitochondrial membranes [96–98]. Therefore, MFN2 may play an essential role in activating innate immune pathways at MAMs and other specialized subcellular regions.

A recent study showed that the energy sensor AMP-activated protein kinase directly interacted with MFN2, inducing autophagy in response to energy stress [99]. Celada et al. confirmed that MFN2 was essential for immune system activation during bacterial infection. MFN2 is required for mitochondrial respiration and the production of ROS. MFN2-mediated ROS production promotes the secretion of cytokines and nitric oxide, induces autophagy and apoptosis, and enhances antigen processing. Myeloid-specific MFN2 deficiency impairs immune responses during infection with Listeria and *M. tuberculosis* and during lipopolysaccharide (LPS)-induced septic shock [12,100]. In accordance with these findings, our recent data suggest that myeloid MFN2 is required for inflammatory and antimicrobial responses during infection with Listeria or *M. tuberculosis* [14]. MFN2-mediated antimicrobial responses during *M. tuberculosis* infection were partly due to HIF-1α-induced xenophagy [14]. On the other hand, MFN2 silencing enhanced intracellular *M. tuberculosis* growth in macrophages [101], presumably due to differences in experimental conditions. These data strongly indicate that MFN2 is a key player in antibacterial innate and adaptive immune responses by coordinating metabolism. Multiple functions of MFN2 in innate immune responses during bacterial infection are summarized in Table 1.

### MFN2 and innate immune responses during parasitic infection

*Leishmania donovani* infection reduces MFN2 expression levels in host cells, associated with mitochondrial depolarization, decreased mitochondrial dynamics, and reduced turnover of microRNA ribonucleoprotein (miRNP) complexes (Summarized in Table 1) [13]. Decreased MFN2 levels may enhance the stabilization of ER-associated miRNPs because MFN2 is involved in the juxtaposition of the ER and mitochondria [13]. Increased miRNP stability downregulates various cytokines, inhibiting pro-inflammatory responses during *Leishmania donovani* infection [13].

## The role of MFN2 in inflammatory and pathological responses

### MFN2 and inflammatory responses

Functional analysis of MFN2 levels in macrophages upon antimicrobial and inflammatory responses revealed that myeloid deficiency of MFN2 but not MFN1 inhibited the production of pro-inflammatory cytokines and innate effector molecules (e.g., nitric oxide) [12]. Additionally, MFN2 deficiency in macrophages suppressed innate immune responses, including autophagy, apoptotic cell clearance, and antigen processing [12,14]. MFN2 was also found to be essential for the induction of strong inflammatory responses in response to LPS and non-septic stimuli [12]. However, the role of MFN2 in sterile inflammation remains unclear.

It is well known that low doses of LPS can lead to low-grade chronic inflammatory diseases. Interestingly, super-low doses of LPS lead to MFN1 ubiquitination and degradation, as well as trigger cell necroptosis due to DRP1 activation. Super-low doses of LPS can activate receptor-interacting protein 3 kinase, which is involved in the assembly of the necrosome complex [102]. Although it remains unclear whether MFN2 degradation is due to the super-low dose of LPS, these data strongly suggest that mitochondria-shaping proteins are fundamental for the crosstalk between host inflammatory responses and intracellular necroptosis [102].

A recent study showed that the orexigenic hormone ghrelin increased MMP and ameliorated LPS-induced glycolysis in macrophages. Interestingly, ghrelin-induced IL-12 secretion was dependent on mitochondria elongation and MFN2. Thus, MFN2-mediated mitochondrial fusion may partly mediate the effects of ghrelin on IL-12 production in macrophages [103]. In a recent study, treatment with a mitochondrial fusion promoter significantly increased MFN2 levels and prevented mitochondrial dysfunction, macrophage infiltration, and apoptosis; these events contributed to reduced brain damage in cardiac ischemia/reperfusion injury [16]. However, the molecular mechanisms regulating MFN2 levels remain largely uncharacterized. Activation of the protein kinase C-α (PKC-α)/heme oxygenase-1 (HO-1) pathway increased the expression levels and superoxide dismutase activities of MFN1 and MFN2. However, the PKC-α/HO-1 axis decreased oxidative stress in response to LPS in rat alveolar macrophages [104]. Future studies are needed to identify the upstream regulators of MFN2 that link mitochondrial dynamics, inflammatory responses, and oxidative stress during sepsis and other inflammatory diseases. The roles of MFN2 in controlling inflammatory responses are summarized in Table 2.

**Table 2.** The role of MFN2 in controlling inflammation, fibrosis, and atherogenesis

Effects	Model	Mechanism	Ref
***Inflammation***			
Inhibition of metabolic-induced inflammation	BMDMs from *Mfn2*-cKO mice	Enhancement of IL-12 secretion by ghrelin	[103]
Alleviation of blood-brain barrier breakdown	Cardiac ischemia/reperfusion injured rats	Increase in claudin5 by pretreatment of mitochondrial fusion promotor M1	[16]
Suppression of LPS-induced mitochondrial dynamic disequilibrium	Murine NR8383 macrophage cells	Restoration of MFN1, MFN2, and OPA1 by activation of PKC-α/HO-1 signaling	[104]
Relieving mitochondrial dysfunction in nonalcoholic fatty liver disease and sepsis	BLN.CL2 hepatocytes; Human liver tissue; *Trem2^−/-^* mice	Decrease in a blockade MFN2 of *miR-106b-5p* through TREM2-mediated exosome	[105]
***Fibrosis***			
Alleviation of liver fibrosis	AML12 hepatocytes; BMDMs; CCl_4_-induced liver fibrosis mice; HSC-T16 hepatic stellate cells	Suppression of TGF-β1/Smad signaling and collagen production	[106]
Protection from kidney fibrosis	*Mfn2*-cKO, *PINK1^−/-^, Prkn^−/-^* mice; BMDMs; Peritoneal macrophages; Renal macrophages	Enhancement of mitophagy through PINK1-mediated MFN2 phosphorylation and recruiting Parkin	[107]
***Atherogenesis***			
Inhibition of intracellular lipid accumulation	*ApoE^−/-^* mice; RAW264.7 and THP-1 cells	Induction of cholesterol transporters by PPARγ activation and inhibition of ERK1/2 and p38	[108]
Augmentation of mitophagy in oxidized LDL-exposed status	*Apoa1bp^−/-^* mice; BMDMs; HEK 293T cells; Human carotid tissue	Ubiquitination of MFN2 through interaction between Parkin and N-terminal domain of AIBP	[17]

AIBP, apolipoprotein A-I binding protein (encoded by *Apoa1bp* gene); ApoE, apolipoprotein E; BMDM, bone marrow-derived macrophage; ERK1/2, extracellular signal-regulated protein kinase 1/2; HO-1, heme oxygenase-1; IL-12, interleukin 12; LDL, low-density lipoprotein; LPS, lipopolysaccharides; MFN2, mitofusin2; *Mfn2*-cKO mice, *Mfn2^fl/fl^:LysM^c/c^* conditional knock-out mice; OPA1, optic atrophy 1 (mitochondrial dynamin like GTPase); PINK1, phosphatase and tensin homolog-induced kinase 1; PKC-α, protein kinase C-alpha; PPARγ, peroxisome proliferator-activated receptor gamma; TGF-β1, transforming growth factor beta 1; TREM2, triggering receptor expressed on myeloid cells 2

### MFN2 and fibrotic responses

Mounting evidence suggests that MFN2 regulates profibrotic responses by modulating the function of macrophages. Hepatic macrophage deficiency due to alterations in TREM2 impaired hepatocytic mitochondria and energy production through the release of exosomes containing the MFN2-targeting micro RNA (miRNA) miR-106b-5p. TREM2 deficiency in mice accelerated the progression of nonalcoholic fatty liver disease (NAFLD) and increased the risk of sepsis [105]. MFN2 inhibits transforming growth factor-beta 1 (TGF-β1)/Smad signaling and the formation of type I, type III, and type IV collagen, thereby ameliorating liver fibrosis and suppressing immune cell infiltration [106]. Hence, MFN2 prevents liver inflammation, fibrosis, and sepsis and is a promising therapeutic target for liver diseases. Moreover, MFN2-deficient macrophages have been shown to promote kidney fibrosis [107]. Future studies are warranted to clarify the molecular mechanisms by which MFN2 regulates fibrotic signals in different tissues and organs. The roles of MFN2 during fibrotic responses are summarized in Table 2.

### MFN2 and atherogenic responses

MFN2 appears to inhibit atherosclerotic plaque formation and vascular inflammation by inhibiting extracellular signal-regulated kinase 1/2 and p38 mitogen-activated protein kinase signaling and upregulating peroxisome proliferator-activated receptor-gamma [108]. Mitochondrial apolipoprotein A-I binding protein (AIBP; encoded by *APOA1BP*) is upregulated in atherosclerotic lesions and forms a complex with E3 ubiquitin-protein ligase PARK2 (Parkin), MFN1, and MFN2. AIBP induces autophagy by regulating MFN1 and MFN2 ubiquitination [17]. Therefore, MFN2 may represent a potential target to prevent atherogenic and inflammatory disorders by modulating autophagy and apoptosis in immune cells. The roles of MFN2 in atherogenic responses are summarized in Table 2.

## MFN2 links immunometabolism to impact immune responses

Emerging evidence demonstrates the converging roles of mitochondrial dynamics and metabolism to regulate inflammatory and immune functions in various immune cells. For example, activated effector T cells have more fragmented mitochondria, whereas memory T cells maintain fused forms to influence specific characteristics in immunometabolic reprogramming, which is connected to distinct profiles of immune functions [58,109,110]. Conversely, differential metabolic cues also can control the mitochondrial dynamics to acquire specific morphologies that impact the immune functions [56,110]. Clearly, there is a reciprocal relationship between mitochondrial metabolism and morphologies to finely tune the optimal immune responses to and protect cells from infectious and inflammatory insults. In this section, we focus on the current understanding of MFN2 in cross talks with immune cell metabolism and function.

**Figure 1.**
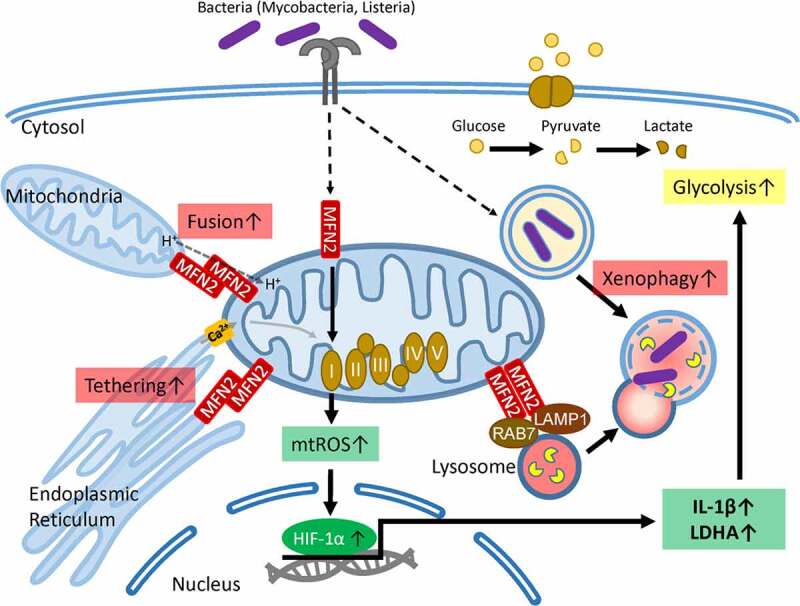
A schematic model of MFN2 and its metabolic roles in macrophages during infection during mycobacterial infection, macrophage MFN2 functions in the enhancement of host defense through activation of inflammatory cytokine generation and xenophagy via interaction with LAMP1. MFN2-mediated innate immune activation depends on the generation of mitochondrial reactive oxygen species (ROS), which are mainly produced by mitochondrial respiratory chain complex I. mitochondrial ROS are required for the induction of HIF-1α that leads to the production of IL-1β for inflammatory signaling and gearing aerobic glycolysis up by LDHA. Additionally, MFN2 is crucial for the maintenance of mitochondrial Ca^2+^ homeostasis and mitochondrial membrane potential through tethering mitochondria and endoplasmic reticulum. HIF-1α, hypoxia-induced factor 1-α; IL-1β, interleukin 1 beta; LAMP1, lysosomal-associated membrane protein 1; LDHA, lactate dehydrogenase A; MFN2, mitofusion2

We have recently shown that MFN2-induced HIF-1α activation in macrophages is required for xenophagy activation, aerobic glycolysis, and inflammatory responses in response to infectious agents (Figure 1) [14]. HIF-1α is a key transcription factor regulating aerobic glycolysis, immunometabolic reprogramming, and M1 polarization in macrophages [111,112]. Although MFN2 deficiency does not affect baseline glycolysis in macrophages [12], it is required for the induction of aerobic glycolysis during *M. tuberculosis* infection [14]. Intriguingly, MFN2-mediated HIF-1α activation is augmented by mitochondrial respiratory chain complex I and ROS and contributes to macrophage-mediated inflammatory responses during infection [14]. Thus, MFN2 activation in macrophages is critical for the induction of inflammation in response to diverse stimuli. The MFN2-induced aerobic glycolysis during infection is at least partly mediated by HIF-1α. However, it remains unclear why MFN2 but not MFN1 is required for immunometabolic remodeling in macrophages during infection. Furthermore, the mechanisms underlying mitochondrial respiratory chain complex I downregulation in MFN2-deficient macrophages remain elusive [14]. Although most evidence on the role of MFN2 in innate immunity is derived from mouse models, it is tempting to speculate that MFN2 plays a similar protective function in humans. Elucidation of the MFN2-mediated signaling networks will not only provide important insights into metabolic rewiring in innate immune cells but also uncover therapeutic targets to treat infections.

## Conclusion

Accumulating evidence shows that mitochondrial dynamics and innate immune responses communicate and regulate each other to influence the infection outcome. It seems that a coordinated interaction between the mitochondrial network and the innate immune signal is vital for host defense. However, dysregulated mitochondrial architecture and function may lead to aberrant immunometabolism, chronic inflammation, and tissue damage, resulting in poor infection outcomes. Therefore, the crosstalk between mitochondrial dynamics and innate immunity must be finely controlled to induce optimal antimicrobial responses while preventing unwanted tissue-destructive inflammation. In this regard, it is crucial to mechanistically characterize the roles of each component of mitochondrial fusion and fission during infection and inflammation.

In this article, we highlight the fine-tuning role of MFN2 in host responses against various infections and inflammatory stimuli. MFN2 appears to be a key regulator of innate immunity, as it interacts with MAVS, NLRP3, and various other signaling molecules during viral infections. Furthermore, MFN2 contributes to macrophage-mediated antibacterial responses, including phagocytosis, inflammation, and xenophagy. As MFN2 orchestrates inflammatory, atherogenic, and fibrotic responses, it may represent a promising target for the treatment of inflammatory and pathological conditions. Although a previous study showed that single nucleotide polymorphisms in *MFN2* were not associated with the risk of leprosy in Chinese patients [113], clinical data on the role of MFN2 in infectious diseases are limited. Furthermore, the pathophysiological roles of MFN2 in human infectious diseases will need to be characterized in future clinical studies. Targeting MFN2 to modulate the balance between innate immunity and pathogenic inflammation may be a worthwhile therapeutic strategy for various infectious and inflammatory diseases.

## Data Availability

Data sharing not applicable – no new data generated.

## References

[1] Harner ME, Unger AK, Geerts WJ, et al. An evidence based hypothesis on the existence of two pathways of mitochondrial crista formation. Elife. 2016;5:e18853. 10.7554/eLife.18853 PMC5138035 27849155

[2] Rojo M, Legros F, Chateau D, et al. Membrane topology and mitochondrial targeting of mitofusins, ubiquitous mammalian homologs of the transmembrane GTPase Fzo. J Cell Sci. 2002;115(8):1663–1674. 10.1242/jcs.115.8.1663 11950885

[3] Misaka T, Miyashita T, Kubo Y. Primary structure of a dynamin-related mouse mitochondrial GTPase and its distribution in brain, subcellular localization, and effect on mitochondrial morphology. J Biol Chem. 2002;277(18):15834–15842. 11847212 10.1074/jbc.M109260200

[4] Li M, Guo J, Wang H, et al. Involvement of Mitochondrial Dynamics and Mitophagy in Sevoflurane-Induced Cell Toxicity. Oxid Med Cell Longev. 2021;2021:6685468. 33728028 10.1155/2021/6685468 PMC7937461

[5] Loson OC, Song Z, Chen H, et al. Fis1, Mff, MiD49, and MiD51 mediate Drp1 recruitment in mitochondrial fission. Mol Biol Cell. 2013;24(5):659–667. 23283981 10.1091/mbc.E12-10-0721 PMC3583668

[6] Xian H, Liou YC. Functions of outer mitochondrial membrane proteins: mediating the crosstalk between mitochondrial dynamics and mitophagy. Cell Death Differ. 2021;28(3):827–842. 33208889 10.1038/s41418-020-00657-z PMC7937681

[7] Chan DC. Fusion and fission: interlinked processes critical for mitochondrial health. Annu Rev Genet. 2012;46(1):265–287. 22934639 10.1146/annurev-genet-110410-132529

[8] Castanier C, Garcin D, Vazquez A, et al. Mitochondrial dynamics regulate the RIG-I-like receptor antiviral pathway. EMBO Rep. 2010;11(2):133–138. 20019757 10.1038/embor.2009.258 PMC2828750

[9] Kapetanovic R, Afroz SF, Ramnath D, et al. Lipopolysaccharide promotes Drp1-dependent mitochondrial fission and associated inflammatory responses in macrophages. Immunol Cell Biol. 2020;98(7):528–539. 10.1111/imcb.12363 PMC7497224 32686869

[10] Gasteiger G, D’Osualdo A, Schubert DA, et al. Cellular Innate Immunity: an Old Game with New Players. J Innate Immun. 2017;9(2):111–125. 28006777 10.1159/000453397 PMC6738785

[11] Yasukawa K, Oshiumi H, Takeda M, et al. Mitofusin 2 inhibits mitochondrial antiviral signaling. Sci Signal. 2009;2(84):ra47. 19690333 10.1126/scisignal.2000287

[12] Tur J, Pereira-Lopes S, Vico T, et al. Mitofusin 2 in Macrophages Links Mitochondrial ROS Production, Cytokine Release, Phagocytosis, Autophagy, and Bactericidal Activity. Cell Rep. 2020;32(8):108079. 32846136 10.1016/j.celrep.2020.108079

[13] Chakrabarty Y, Bhattacharyya SN. Leishmania donovani restricts mitochondrial dynamics to enhance miRNP stability and target RNA repression in host macrophages. Mol Biol Cell. 2017;28(15):2091–2105. 28539410 10.1091/mbc.E16-06-0388 PMC5509422

[14] Silwal P, Kim JK, Jeon SM, et al. Mitofusin-2 boosts innate immunity through the maintenance of aerobic glycolysis and activation of xenophagy in mice. Commun Biol. 2021;4(1):548. 33972668 10.1038/s42003-021-02073-6 PMC8110749

[15] Ballard A, Zeng R, Zarei A, et al. The tethering function of mitofusin2 controls osteoclast differentiation by modulating the Ca(2+)-NFATc1 axis. J Biol Chem. 2020;295(19):6629–6640. 32165499 10.1074/jbc.RA119.012023 PMC7212632

[16] Surinkaew P, Apaijai N, Sawaddiruk P, et al. Mitochondrial fusion promoter alleviates brain damage in rats with cardiac ischemia/reperfusion injury. J Alzheimers Dis. 2020;77(3):993–1003. 32804148 10.3233/JAD-200495

[17] Choi SH, Agatisa-Boyle C, Gonen A, et al. Intracellular AIBP (Apolipoprotein A-I Binding Protein) Regulates Oxidized LDL (Low-Density Lipoprotein)-Induced Mitophagy in Macrophages. Arterioscler Thromb Vasc Biol. 2021;41(2):e82–e96. 33356389 10.1161/ATVBAHA.120.315485 PMC8105271

[18] Tosi MF. Innate immune responses to infection. J Allergy Clin Immunol. 2005;116(2):241–249. quiz 50. 16083775 10.1016/j.jaci.2005.05.036

[19] Blach-Olszewska Z. Innate immunity: cells, receptors, and signaling pathways. Arch Immunol Ther Exp (Warsz). 2005;53:245–253. 15995585

[20] Iwasaki A, Medzhitov R. Toll-like receptor control of the adaptive immune responses. Nat Immunol. 2004;5(10):987–995. 10.1038/ni1112 15454922

[21] Marshall JS, Warrington R, Watson W, et al. An introduction to immunology and immunopathology. Allergy Asthma Clin Immunol. 2018;14(S2):49. 30263032 10.1186/s13223-018-0278-1 PMC6156898

[22] Warrington R, Watson W, Kim HL, et al. An introduction to immunology and immunopathology. Allergy Asthma Clin Immunol. 2011;7(Suppl 1):S1. 22165815 10.1186/1710-1492-7-S1-S1 PMC3245432

[23] Diskin C, Palsson-McDermott EM. Metabolic Modulation in Macrophage Effector Function. Front Immunol. 2018;9:270. 29520272 10.3389/fimmu.2018.00270 PMC5827535

[24] Van den Bossche J, O’Neill LA, Menon D. Macrophage Immunometabolism: where Are We (Going)? Trends Immunol. 2017;38(6):395–406. 28396078 10.1016/j.it.2017.03.001

[25] O’Neill LA, Pearce EJ. Immunometabolism governs dendritic cell and macrophage function. J Exp Med. 2016;213(1):15–23. 26694970 10.1084/jem.20151570 PMC4710204

[26] Ablasser A, Hur S. Regulation of cGAS- and RLR-mediated immunity to nucleic acids. Nat Immunol. 2020;21(1):17–29. 31819255 10.1038/s41590-019-0556-1

[27] Kell AM, Gale M Jr. RIG-I in RNA virus recognition. Virology. 2015;479-480:110–121. 25749629 10.1016/j.virol.2015.02.017 PMC4424084

[28] Hou F, Sun L, Zheng H, et al. MAVS forms functional prion-like aggregates to activate and propagate antiviral innate immune response. Cell. 2011;146(3):448–461. 21782231 10.1016/j.cell.2011.06.041 PMC3179916

[29] Yoshinaka T, Kosako H, Yoshizumi T, et al. Structural Basis of Mitochondrial Scaffolds by Prohibitin Complexes: insight into a Role of the Coiled-Coil Region. iScience. 2019;19:1065–1078. 31522117 10.1016/j.isci.2019.08.056 PMC6745515

[30] Hou J, Han L, Zhao Z, et al. USP18 positively regulates innate antiviral immunity by promoting K63-linked polyubiquitination of MAVS. Nat Commun. 2021;12(1):2970. 34016972 10.1038/s41467-021-23219-4 PMC8137702

[31] Dai T, Wu L, Wang S, et al. FAF1 Regulates Antiviral Immunity by Inhibiting MAVS but Is Antagonized by Phosphorylation upon Viral Infection. Cell Host Microbe. 2018;24(6):776–90 e5. 30472208 10.1016/j.chom.2018.10.006

[32] Wies E, Wang MK, Maharaj NP, et al. Dephosphorylation of the RNA sensors RIG-I and MDA5 by the phosphatase PP1 is essential for innate immune signaling. Immunity. 2013;38(3):437–449. 23499489 10.1016/j.immuni.2012.11.018 PMC3616631

[33] Oshiumi H, Matsumoto M, Seya T. Ubiquitin-mediated modulation of the cytoplasmic viral RNA sensor RIG-I. J Biochem. 2012;151(1):5–11. 21890623 10.1093/jb/mvr111

[34] Zeng W, Sun L, Jiang X, et al. Reconstitution of the RIG-I pathway reveals a signaling role of unanchored polyubiquitin chains in innate immunity. Cell. 2010;141(2):315–330. 20403326 10.1016/j.cell.2010.03.029 PMC2919214

[35] Gack MU, Shin YC, Joo CH, et al. TRIM25 RING-finger E3 ubiquitin ligase is essential for RIG-I-mediated antiviral activity. Nature. 2007;446(7138):916–920. 17392790 10.1038/nature05732

[36] Eisenacher K, Krug A. Regulation of RLR-mediated innate immune signaling–it is all about keeping the balance. Eur J Cell Biol. 2012;91(1):36–47. 21481967 10.1016/j.ejcb.2011.01.011

[37] Schmidt A, Rothenfusser S, Hopfner KP. Sensing of viral nucleic acids by RIG-I: from translocation to translation. Eur J Cell Biol. 2012;91(1):78–85. 21496944 10.1016/j.ejcb.2011.01.015 PMC3155743

[38] Elesela S, Lukacs NW. Role of Mitochondria in Viral Infections. Life (Basel). 2021;11(3):232. 10.3390/life11030232 PMC7998235 33799853

[39] Khan M, Syed GH, Kim SJ, et al. Mitochondrial dynamics and viral infections: a close nexus. Biochim Biophys Acta. 2015;1853(10):2822–2833. 25595529 10.1016/j.bbamcr.2014.12.040 PMC4500740

[40] Kim SJ, Ahn DG, Syed GH, et al. The essential role of mitochondrial dynamics in antiviral immunity. Mitochondrion. 2018;41:21–27. 29246869 10.1016/j.mito.2017.11.007 PMC5988924

[41] Paik S, Kim JK, Silwal P, et al. An update on the regulatory mechanisms of NLRP3 inflammasome activation. Cell Mol Immunol. 2021;18:1141–1160. 33850310 10.1038/s41423-021-00670-3 PMC8093260

[42] Kv S, Deng M, Jp T. The NLRP3 inflammasome: molecular activation and regulation to therapeutics. Nat Rev Immunol. 2019;19(8):477–489. 31036962 10.1038/s41577-019-0165-0 PMC7807242

[43] Missiroli S, Patergnani S, Caroccia N, et al. Mitochondria-associated membranes (MAMs) and inflammation. Cell Death Dis. 2018;9(3):329. 29491386 10.1038/s41419-017-0027-2 PMC5832426

[44] Thoudam T, Jeon JH, Ha CM, et al. Role of mitochondria-associated endoplasmic reticulum membrane in inflammation-mediated metabolic diseases. Mediators Inflamm. 2016;2016:1851420. 28074080 10.1155/2016/1851420 PMC5198184

[45] Raturi A, Simmen T. Where the endoplasmic reticulum and the mitochondrion tie the knot: the mitochondria-associated membrane (MAM). Biochim Biophys Acta. 2013;1833(1):213–224. 22575682 10.1016/j.bbamcr.2012.04.013

[46] Kelly B, O’Neill LA. Metabolic reprogramming in macrophages and dendritic cells in innate immunity. Cell Res. 2015;25(7):771–784. 26045163 10.1038/cr.2015.68 PMC4493277

[47] Palsson-McDermott EM, O’Neill LA. The Warburg effect then and now: from cancer to inflammatory diseases. Bioessays. 2013;35(11):965–973. 24115022 10.1002/bies.201300084

[48] Mills E, O’Neill LA. Succinate: a metabolic signal in inflammation. Trends Cell Biol. 2014;24(5):313–320. 24361092 10.1016/j.tcb.2013.11.008

[49] Tannahill GM, Curtis AM, Adamik J, et al. Succinate is an inflammatory signal that induces IL-1beta through HIF-1alpha. Nature. 2013;496(7444):238–242. 23535595 10.1038/nature11986 PMC4031686

[50] Ramond E, Jamet A, Coureuil M, et al. Pivotal Role of Mitochondria in Macrophage Response to Bacterial Pathogens. Front Immunol. 2019;10:2461. 31708919 10.3389/fimmu.2019.02461 PMC6819784

[51] Viola A, Munari F, Sanchez-Rodriguez R, et al. The metabolic signature of macrophage responses. Front Immunol. 2019;10:1462. 31333642 10.3389/fimmu.2019.01462 PMC6618143

[52] Batista-Gonzalez A, Vidal R, Criollo A, et al. New Insights on the Role of Lipid Metabolism in the Metabolic Reprogramming of Macrophages. Front Immunol. 2019;10:2993. 31998297 10.3389/fimmu.2019.02993 PMC6966486

[53] Pelgrom LR, Everts B. Metabolic control of type 2 immunity. Eur J Immunol. 2017;47(8):1266–1275. 28661041 10.1002/eji.201646728

[54] Covarrubias AJ, Aksoylar HI, Yu J, et al. Akt-mTORC1 signaling regulates Acly to integrate metabolic input to control of macrophage activation. Elife. 2016;5:e11612. 10.7554/eLife.11612 PMC4769166 26894960

[55] Angajala A, Lim S, Phillips JB, et al. Diverse Roles of Mitochondria in Immune Responses: novel Insights Into Immuno-Metabolism. Front Immunol. 2018;9:1605. 30050539 10.3389/fimmu.2018.01605 PMC6052888

[56] Rambold AS, Pearce EL. Mitochondrial Dynamics at the Interface of Immune Cell Metabolism and Function. Trends Immunol. 2018;39(1):6–18. 28923365 10.1016/j.it.2017.08.006

[57] Mills EL, Kelly B, O’Neill LAJ. Mitochondria are the powerhouses of immunity. Nat Immunol. 2017;18(5):488–498. 28418387 10.1038/ni.3704

[58] Buck MD, O’Sullivan D, Klein Geltink RI, et al. Mitochondrial Dynamics Controls T Cell Fate through Metabolic Programming. Cell. 2016;166(1):63–76. 27293185 10.1016/j.cell.2016.05.035 PMC4974356

[59] Hom J, Sheu SS. Morphological dynamics of mitochondria–a special emphasis on cardiac muscle cells. J Mol Cell Cardiol. 2009;46(6):811–820. 19281816 10.1016/j.yjmcc.2009.02.023 PMC2995918

[60] Griffin EE, Detmer SA, Chan DC. Molecular mechanism of mitochondrial membrane fusion. Biochim Biophys Acta. 2006;1763(5–6):482–489. 16571363 10.1016/j.bbamcr.2006.02.003

[61] Ranieri M, Brajkovic S, Riboldi G, et al. Mitochondrial fusion proteins and human diseases. Neurol Res Int. 2013;2013:293893. 23781337 10.1155/2013/293893 PMC3678461

[62] Xin Y, Li J, Wu W, et al. Mitofusin-2: a New Mediator of Pathological Cell Proliferation. Front Cell Dev Biol. 2021;9:647631. 33869201 10.3389/fcell.2021.647631 PMC8049505

[63] Ishihara N, Eura Y, Mihara K. Mitofusin 1 and 2 play distinct roles in mitochondrial fusion reactions via GTPase activity. J Cell Sci. 2004;117(26):6535–6546. 15572413 10.1242/jcs.01565

[64] Twig G, Elorza A, Molina AJ, et al. Fission and selective fusion govern mitochondrial segregation and elimination by autophagy. EMBO J. 2008;27(2):433–446. 18200046 10.1038/sj.emboj.7601963 PMC2234339

[65] Youle RJ. van der Bliek AM. Mitochondrial fission, fusion, and stress. Science. 2012;337(6098):1062–1065. 22936770 10.1126/science.1219855 PMC4762028

[66] Tondera D, Grandemange S, Jourdain A, et al. SLP-2 is required for stress-induced mitochondrial hyperfusion. EMBO J. 2009;28(11):1589–1600. 19360003 10.1038/emboj.2009.89 PMC2693158

[67] Lee YJ, Jeong SY, Karbowski M, et al. Roles of the mammalian mitochondrial fission and fusion mediators Fis1, Drp1, and Opa1 in apoptosis. Mol Biol Cell. 2004;15(11):5001–5011. 10.1091/mbc.E04-04-0294 PMC524759 15356267

[68] Li C, Liu W, Wang F, et al. DNA damage-triggered activation of cGAS-STING pathway induces apoptosis in human keratinocyte HaCaT cells. Mol Immunol. 2021;131:180–190. 33423764 10.1016/j.molimm.2020.12.037

[69] Yu CH, Davidson S, Harapas CR, et al. TDP-43 Triggers Mitochondrial DNA Release via mPTP to Activate cGAS/STING in ALS. Cell. 2020;183(3):636–49 e18. 33031745 10.1016/j.cell.2020.09.020 PMC7599077

[70] Zhang X, Bai XC, Chen ZJ. Structures and Mechanisms in the cGAS-STING Innate Immunity Pathway. Immunity. 2020;53(1):43–53. 32668227 10.1016/j.immuni.2020.05.013

[71] Hopfner KP, Hornung V. Molecular mechanisms and cellular functions of cGAS-STING signalling. Nat Rev Mol Cell Biol. 2020;21(9):501–521. 32424334 10.1038/s41580-020-0244-x

[72] Kwon D, Park E, Kang SJ. Stimulator of IFN genes-mediated DNA-sensing pathway is suppressed by NLRP3 agonists and regulated by mitofusin 1 and TBC1D15, mitochondrial dynamics mediators. FASEB J. 2017;31(11):4866–4878. 28729291 10.1096/fj.201700328R

[73] Eura Y, Ishihara N, Yokota S, et al. Two mitofusin proteins, mammalian homologues of FZO, with distinct functions are both required for mitochondrial fusion. J Biochem. 2003;134(3):333–344. 10.1093/jb/mvg150 14561718

[74] Filadi R, Pendin D, Pizzo P. Mitofusin 2: from functions to disease. Cell Death Dis. 2018;9(3):330. 29491355 10.1038/s41419-017-0023-6 PMC5832425

[75] Zorzano A, Liesa M, Sebastian D, et al. Mitochondrial fusion proteins: dual regulators of morphology and metabolism. Semin Cell Dev Biol. 2010;21(6):566–574. 20079867 10.1016/j.semcdb.2010.01.002

[76] Ishihara N, Otera H, Oka T, et al. Regulation and physiologic functions of GTPases in mitochondrial fusion and fission in mammals. Antioxid Redox Signal. 2013;19(4):389–399. 22871170 10.1089/ars.2012.4830

[77] Chen Y, Dorn GW 2nd PINK1-phosphorylated mitofusin 2 is a Parkin receptor for culling damaged mitochondria. Science. 2013;340(6131):471–475. 23620051 10.1126/science.1231031 PMC3774525

[78] Tanaka A, Cleland MM, Xu S, et al. Proteasome and p97 mediate mitophagy and degradation of mitofusins induced by Parkin. J Cell Biol. 2010;191(7):1367–1380. 21173115 10.1083/jcb.201007013 PMC3010068

[79] Wang W, Cheng X, Lu J, et al. Mitofusin-2 is a novel direct target of p53. Biochem Biophys Res Commun. 2010;400(4):587–592. 20804729 10.1016/j.bbrc.2010.08.108

[80] Zhang GE, Jin HL, Lin XK, et al. Antitumor effects of Mfn2 in gastric cancer. Int J Mol Sci. 2013;14(7):13005–13021. 23797661 10.3390/ijms140713005 PMC3742171

[81] Zuchner S, Mersiyanova IV, Muglia M, et al. Mutations in the mitochondrial GTPase mitofusin 2 cause Charcot-Marie-Tooth neuropathy type 2A. Nat Genet. 2004;36(5):449–451. 15064763 10.1038/ng1341

[82] Kijima K, Numakura C, Izumino H, et al. Mitochondrial GTPase mitofusin 2 mutation in Charcot-Marie-Tooth neuropathy type 2A. Hum Genet. 2005;116(1–2):23–27. 15549395 10.1007/s00439-004-1199-2

[83] Misko AL, Sasaki Y, Tuck E, et al. Mitofusin2 mutations disrupt axonal mitochondrial positioning and promote axon degeneration. J Neurosci. 2012;32(12):4145–4155. 22442078 10.1523/JNEUROSCI.6338-11.2012 PMC3319368

[84] Hernandez-Alvarez MI, Thabit H, Burns N, et al. Subjects with early-onset type 2 diabetes show defective activation of the skeletal muscle PGC-1{alpha}/Mitofusin-2 regulatory pathway in response to physical activity. Diabetes Care. 2010;33(3):645–651. 20032281 10.2337/dc09-1305 PMC2827524

[85] Chen KH, Guo X, Ma D, et al. Dysregulation of HSG triggers vascular proliferative disorders. Nat Cell Biol. 2004;6(9):872–883. 15322553 10.1038/ncb1161

[86] Chiong M, Cartes-Saavedra B, Norambuena-Soto I, et al. Mitochondrial metabolism and the control of vascular smooth muscle cell proliferation. Front Cell Dev Biol. 2014;2:72. 25566542 10.3389/fcell.2014.00072 PMC4266092

[87] Xu K, Chen G, Li X, et al. MFN2 suppresses cancer progression through inhibition of mTORC2/Akt signaling. Sci Rep. 2017;7(1):41718. 28176801 10.1038/srep41718 PMC5296837

[88] Sasaki O, Yoshizumi T, Kuboyama M, et al. A structural perspective of the MAVS-regulatory mechanism on the mitochondrial outer membrane using bioluminescence resonance energy transfer. Biochim Biophys Acta. 2013;1833(5):1017–1027. 23337771 10.1016/j.bbamcr.2013.01.010

[89] Luo Z, Liu LF, Jiang YN, et al. Novel insights into stress-induced susceptibility to influenza: corticosterone impacts interferon-beta responses by Mfn2-mediated ubiquitin degradation of MAVS. Signal Transduct Target Ther. 2020;5(1):202. 32943610 10.1038/s41392-020-00238-z PMC7499204

[90] Onoguchi K, Onomoto K, Takamatsu S, et al. Virus-infection or 5ʹppp-RNA activates antiviral signal through redistribution of IPS-1 mediated by MFN1. PLoS Pathog. 2010;6(7):e1001012. 20661427 10.1371/journal.ppat.1001012 PMC2908619

[91] Campbell GR, To RK, Spector SA. TREM-1 Protects HIV-1-Infected Macrophages from Apoptosis through Maintenance of Mitochondrial Function. mBio. 2019;10(6):e02638–19. 10.1128/mBio.02638-19 PMC6851287 31719184

[92] Yu CY, Liang JJ, Li JK, et al. Dengue Virus Impairs Mitochondrial Fusion by Cleaving Mitofusins. PLoS Pathog. 2015;11(12):e1005350. 26717518 10.1371/journal.ppat.1005350 PMC4696832

[93] Koshiba T, Yasukawa K, Yanagi Y, et al. Mitochondrial membrane potential is required for MAVS-mediated antiviral signaling. Sci Signal. 2011;4(158):ra7. 21285412 10.1126/scisignal.2001147

[94] Ichinohe T, Yamazaki T, Koshiba T, et al. Mitochondrial protein mitofusin 2 is required for NLRP3 inflammasome activation after RNA virus infection. Proc Natl Acad Sci U S A. 2013;110(44):17963–17968. 10.1073/pnas.1312571110 PMC3816452 24127597

[95] Xu F, Qi H, Li J, et al. Mycobacterium tuberculosis infection upregulates MFN2 expression to promote NLRP3 inflammasome formation. J Biol Chem. 2020;295(51):17684–17697. 10.1074/jbc.RA120.014077 PMC7762945 33454007

[96] Merkwirth C, Langer T. Mitofusin 2 builds a bridge between ER and mitochondria. Cell. 2008;135(7):1165–1167. 19109886 10.1016/j.cell.2008.12.005

[97] Jung S, Kwon JO, Kim MK, et al. Mitofusin 2, a mitochondria-ER tethering protein, facilitates osteoclastogenesis by regulating the calcium-calcineurin-NFATc1 axis. Biochem Biophys Res Commun. 2019;516(1):202–208. 10.1016/j.bbrc.2019.06.017 31204051

[98] Filadi R, Greotti E, Pizzo P. Highlighting the endoplasmic reticulum-mitochondria connection: focus on Mitofusin 2. Pharmacol Res. 2018;128:42–51. 29309902 10.1016/j.phrs.2018.01.003

[99] Hu Y, Chen H, Zhang L, et al. The AMPK-MFN2 axis regulates MAM dynamics and autophagy induced by energy stresses. Autophagy. 2020;17(5):1142–1156. 10.1080/15548627.2020.1749490 PMC8143230 32249716

[100] Lloberas J, Munoz JP, Hernandez-Alvarez MI, et al. Macrophage mitochondrial MFN2 (mitofusin 2) links immune stress and immune response through reactive oxygen species (ROS) production. Autophagy. 2020;16(12):2307–2309. 33171058 10.1080/15548627.2020.1839191 PMC7751645

[101] Lee J, Choi JA, Cho SN, et al. Mitofusin 2-deficiency suppresses mycobacterium tuberculosis survival in macrophages. Cells. 2019;8(11):1355. 10.3390/cells8111355 PMC6912353 31671648

[102] Baker B, Maitra U, Geng S, et al. Molecular and cellular mechanisms responsible for cellular stress and low-grade inflammation induced by a super-low dose of endotoxin. J Biol Chem. 2014;289(23):16262–16269. 24759105 10.1074/jbc.M114.569210 PMC4047395

[103] Correa da Silva F, Aguiar C, Jas P, et al. Ghrelin effects on mitochondrial fitness modulates macrophage function. Free Radic Biol Med. 2019;145:61–66. 31525456 10.1016/j.freeradbiomed.2019.09.012

[104] Li X, Zhang Y, Yu J, et al. Activation of protein kinase C-alpha/heme oxygenase-1 signaling pathway improves mitochondrial dynamics in lipopolysaccharide-activated NR8383 cells. Exp Ther Med. 2018;16:1529–1537. 30112072 10.3892/etm.2018.6290 PMC6090414

[105] Hou J, Zhang J, Cui P, et al. TREM2 sustains macrophage-hepatocyte metabolic coordination in nonalcoholic fatty liver disease and sepsis. J Clin Invest. 2021;131(4):e135197. 10.1172/JCI135197 PMC7880419 33586673

[106] Zhu H, Shan Y, Ge K, et al. Specific Overexpression of Mitofusin-2 in Hepatic Stellate Cells Ameliorates Liver Fibrosis in Mice Model. Hum Gene Ther. 2020;31(1–2):103–109. 31802713 10.1089/hum.2019.153

[107] Bhatia D, Chung KP, Nakahira K, et al. Mitophagy-dependent macrophage reprogramming protects against kidney fibrosis. JCI Insight. 2019;4(23):e132826. 10.1172/jci.insight.132826 PMC6962025 31639106

[108] Liu C, Ge B, He C, et al. Mitofusin 2 decreases intracellular lipids in macrophages by regulating peroxisome proliferator-activated receptor-gamma. Biochem Biophys Res Commun. 2014;450(1):500–506. 24928385 10.1016/j.bbrc.2014.06.005

[109] Lanna A, Dustin ML. Mitochondrial fusion fuels T cell memory. Cell Res. 2016;26(9):969–970. 27514702 10.1038/cr.2016.94 PMC5034112

[110] Wai T, Langer T. Mitochondrial Dynamics and Metabolic Regulation. Trends Endocrinol Metab. 2016;27(2):105–117. 26754340 10.1016/j.tem.2015.12.001

[111] Corcoran SE, O’Neill LA. HIF1alpha and metabolic reprogramming in inflammation. J Clin Invest. 2016;126(10):3699–3707. 27571407 10.1172/JCI84431 PMC5096812

[112] Prigione A, Rohwer N, Hoffmann S, et al. HIF1alpha modulates cell fate reprogramming through early glycolytic shift and upregulation of PDK1-3 and PKM2. Stem Cells. 2014;32(2):364–376. 24123565 10.1002/stem.1552 PMC5730046

[113] Wang D, Li GD, Zhang DF, et al. Genetic variants of the MAVS, MITA and MFN2 genes are not associated with leprosy in Han Chinese from Southwest China. Infect Genet Evol. 2016;45:105–110. 27553710 10.1016/j.meegid.2016.08.021

